# A nationwide analysis of population group differences in the COVID-19 epidemic in Israel, February 2020–February 2021

**DOI:** 10.1016/j.lanepe.2021.100130

**Published:** 2021-06-05

**Authors:** Khitam Muhsen, Wasef Na'aminh, Yelena Lapidot, Sophy Goren, Yonatan Amir, Saritte Perlman, Manfred S. Green, Gabriel Chodick, Dani Cohen

**Affiliations:** a Department of Epidemiology and Preventive Medicine, School of Public Health, Sackler Faculty of Medicine, Tel Aviv University, Tel Aviv, 6139001, Israel; b University of Haifa, School of Public Health, Haifa, Israel; c Maccabi Institute for Research & Innovation, Maccabi Healthcare Services, Kaufman 4, Tel Aviv, Israel

**Keywords:** SARS-CoV-2, Incidence, Mortality, Israel, Minority, Social determinants, BNT162b2 vaccine, Immunisation uptake

## Abstract

**Background:**

Social inequalities affect the COVID-19 burden and vaccine uptake. The aim of this study was to explore inequalities in the incidence and mortality rate of SARS-CoV-2 infection and vaccine uptake in various sociodemographic and population group strata in Israel.

**Methods:**

We analysed nationwide publicly available, aggregated data on PCR-confirmed SARS-CoV-2 infections and COVID-19 deaths between March 2020 and February 2021, as well as the first three months of COVID-19 immunisation according to sociodemographics, including population group and residential socioeconomic status (SES). We computed incidence and mortality rates of COVID-19. Comparisons between towns with predominantly Arab, ultra-Orthodox Jewish (the minorities), general Jewish populations, and according to SES, were conducted using generalised linear models with negative binomial distribution.

**Findings:**

Overall, 774,030 individuals had SARS-CoV-2 infection (cumulative incidence 84•5 per 1,000 persons) and 5687 COVID-19 patients had died (mortality rate 62•8 per 100,000 persons). The highest mortality rate was found amongst the elderly. Most (>75%) individuals aged 60 years or above have been vaccinated with BNT162b2 vaccine. The risk of SARS-CoV-2 infection was higher in towns with predominantly Arab and ultra-Orthodox Jewish populations than in the general Jewish population, and in low SES communities. COVID-19 mortality rate was highest amongst Arabs. Conversely, vaccine uptake was lower amongst Arab and ultra-Orthodox Jewish populations and low SES communities.

**Interpretation:**

Ethnic and religious minorities and low SES communities experience substantial COVID-19 burden, and have lower vaccine uptake, even in a society with universal accessibility to healthcare. Quantifying these inequalities is fundamental towards reducing these gaps, which imposes a designated apportion of resources to adequately control the pandemic.

**Funding:**

No external funding was available for this study.

**Research in context**

**Evidence before this study**

We searched PubMed without any language restrictions using the following search terms: “social disparities” OR “COVID-19″ OR “mortality” OR “incidence” OR “SARS-CoV-2″ OR “vaccines” until March 17, 2021. A few studies, mainly from the United States, the United Kingdom and Sweden, reported social inequalities in COVID-19 mortality; we did not find studies on social inequalities in COVID-19 vaccine uptake.

**Added value of this study**

Complex social inequalities in the COVID-19 burden were identified in Israel, with higher incidence rates in the Arab population, the main ethnic minority, and the ultra-Orthodox Jewish population, a religious minority, both of low socioeconomic status (SES) compared to the general Jewish population. Moreover, higher incidence rates were found in low versus high socioeconomic communities, irrespective of minority status. The risk of COVID-19 mortality was higher in the Arab population. Despite the remarkable achievement of vaccinating more than half of the population with the BNT162b2 COVID-19 vaccine within three months, vaccine uptake was lower amongst Arabs, ultra-Orthodox Jews and low socioeconomic communities. Remarkably, these inequalities were evident even in settings with universal health insurance and high accessibility to healthcare, including COVID-19 diagnostics, treatment and immunisation, like Israel. Our study raises awareness of these disparities and encourages the understanding of their roots in order to better mitigate the impact of the COVID-19 pandemic.

**Implications of all the available evidence**

Pre-existing social inequalities were major drivers of the disparity in the COVID-19 burden and vaccine uptake in Israel. Quantifying the role of social determinants in the COVID-19 burden and vaccine uptake is essential for closing these gaps, which necessitates additional allocation of resources. To achieve equitable control of the pandemic, interventions to reduce social disparities in COVID-19 should be enhanced amongst ethnic and religious minorities and low SES communities, including providing educational programs in the sub-population's language, enhancing accessibility to vaccines and improving trust in policy makers.

**Role of the funding source**

There was no external funding for this study.

Alt-text: Unlabelled box

## 1 Introduction

Coronavirus disease 2019 (COVID-19), caused by severe acute respiratory syndrome coronavirus 2 (SARS-CoV-2) [1], resulted in an enormous health, economic and societal burden worldwide [1]. Variations in COVID-19 mortality rates across countries are partially explained by demographics, including population density, proportion of people aged 80 years and above, age distribution of the cases, urban population and domestic growth product [2,3]. In some countries, there is evidence of social and ethnic disparities in the burden of COVID-19 [4], [5], [6]. In December 2020 and the beginning of 2021, several COVID-19 vaccines were licensed or received emergency use authorisation in numerous countries, which opened a new era in pandemic control. Nevertheless, vaccine hesitancy remains an issue, with differences between sub-populations in intention to receive COVID-19 vaccines, for example, according to ethnicity, sex and age groups and educational levels, as shown in surveys conducted before COVID-19 vaccines became available [7,8].

Israel has a population of more than nine million people with diverse ethnic and cultural sub-populations. Approximately 74 percent are Jews, 21% are Arabs and 5% belong to other ethnicities [9]. In the Jewish population, about 12% belong to a distinct subpopulation which is religiously ultra-Orthodox [10], and is characterised by a lower rate of secular education. Both the Arab and ultra-Orthodox Jewish populations are of lower socioeconomic status (SES), have higher fertility rates and are younger compared to the general Jewish population [10], [11], [12], [13]. Typically, these population groups live in different towns or neighbourhoods. All Israelis have universal health insurance under the National Health Insurance Law [14]. This covers outpatient and inpatient healthcare and includes PCR testing for SARS-CoV-2 and COVID-19-related treatments and immunisation.

In general, there are disparities in infectious disease incidence and vaccine uptake according to population group. For example, large measles outbreaks have occurred significantly more commonly in the ultra-Orthodox Jewish communities due to low immunisation coverage [15,16]. The vaccination uptake of routine childhood vaccines is highest in the Arab population. However, influenza vaccine uptake is lower in both the Arab and ultra-Orthodox Jewish populations compared to the general Jewish population [17].

During the early phases of the pandemic, the efforts of the Israeli Ministry of Health (MOH) focused on preparedness and preventing the importation of COVID-19 into the country, including introducing international travel restrictions, quarantine for people who returned from abroad [18] and establishment of molecular testing [18]. On February 21, 2020, the first COVID-19 case was detected in Israel in a woman who had returned from Japan and had been quarantined on the *Diamond Princess* cruise liner. Shortly thereafter, community transmission was established [19,20]. Travel-related and community transmission was demonstrated by full viral genome sequences [21]. In parallel, designated departments for treating patients with COVID-19 were opened in all hospitals. Training sessions were organised for medical staff on treatment protocols using personal protective equipment and infection control procedures [18]. As of February 2021, there were 774,030 PCR-confirmed SARS-CoV-2 cases in Israel.

Non-pharmaceutical preventive measures were implemented to control the epidemic **(Supplementary Table 1)**, which included contact tracing (via epidemiological investigation and digital tracing), and quarantine for close contacts of confirmed cases. Restrictions on mobility and gatherings were introduced; wearing of face masks in public areas became compulsory by law; and campaigns were run in various languages on the importance of hygiene and physical distancing. A number of countrywide lockdowns were also imposed. On December 19, 2020, Israel introduced the BNT162b2 mRNA vaccine [22] in a vigorous immunisation campaign.

Given the rapidly evolving COVID-19 epidemic globally and the emergence of evidence suggesting social inequalities in COVID-19 incidence and mortality [4], [5], [6], the aim of this study was to assess inequalities in the incidence and mortality rates related to SARS-CoV-2 infection and vaccine uptake in various socio-demographic and population strata in Israel. In particular, we aimed to document the risk for COVID-19 across sub-population such as secular and traditionally observant Jews, ultra-Orthodox Jews and Arabs. This nationwide analysis provides essential insight on SARS-CoV-2-related social disparities and a baseline for COVID-19 vaccine impact and effectiveness assessments.

## 2 Methods

### 2.1 Study design and population

This descriptive study was undertaken using nationwide publicly available data on SARS-CoV-2 infection in Israel.

### 2.2 Data sources

We used the following sources:

1. We accessed publicly available aggregated data reported by the Israel Ministry of Health (MOH) [23] on the number of PCR-confirmed SARS-CoV-2 cases between March 15, 2020 and February 27, 2021 and the number of COVID-19 hospitalisations and COVID-19 deaths. Information was collected on COVID-19 immunisation between December 19, 2020 and March 2, 2021. Data are reported in an aggregate manner: overall and by age group, sex and time. Information was also obtained at the level of town/city of residence. These data are uploaded to the Israel MOH website in several datasets that are updated frequently.
2. We used the databases of the Israel Central Bureau of Statistics (ICBS) [9] to obtain information on the sizes of the overall and sub-populations size, and the results from national surveys on public adherence to COVID-19 non-pharmaceutical preventive measures. Adherence was defined based on the self-reports of the participants in these surveys in response to the questions on the degree to which they complied with the Israel MOH guidelines of maintaining two-metre physical distancing between people in public settings, wearing a mask, and personal hygiene such as hand washing and use of hand sanitiser.

## 3 Study variables

### 3.1 Case definition

A confirmed COVID-19 case was defined as having a positive PCR test. In the initial phase of the epidemic in Israel, SARS-CoV-2 PCR testing was limited to individuals with an epidemiological link to cases of COVID-19 and symptoms consistent with COVID-19. Early in the epidemic, testing was not offered to asymptomatic contacts of COVID-19 patients. By June 2020, PCR testing was offered to contacts of infected people regardless of symptoms. The rationale of this change was the identification of individuals with asymptomatic SARS-CoV-2 infection who potentially could transmit the virus and maximising the number of people who should be in quarantine. Drive-in stations for specimen collection were established across the country, including mobile stations to increase accessibility and to reach remote areas. These actions resulted in a continuous rise in test utilisation **(Supplementary Figure 1).**

### 3.2 COVID-19 immunisation

Vaccination against COVID-19 began in Israel on December 19, 2020 using the BNT162b2 vaccine, prioritising healthcare workers, persons aged 60 years or older and those with underlying conditions. COVID-19 immunisation has been expanded to include individuals aged 16 years and over. The vaccine is given to eligible individuals free of charge in two doses three weeks apart. Immunisation is carried out through immunisation centres run by the Health Maintenance Organizations (HMOs), and with the aid of the Home Front Command, Magen David Adom and local municipalities to increase accessibility and vaccine uptake in nursing homes and remote areas. Immunisation is documented in a national database and is entered into the person's electronic health record held by the health fund insuring them.

### 3.3 Socio-demographics

Age and sex were defined based on the groups determined in the MOH database [23]. The median age in each residential town was obtained from the ICBS [24].

SES was determined using the ICBS classification of the residential SES rank at the level of city/town/village. Ranks are on a scale from one to 10, with lower ranks representing a lower SES. This is an aggregate SES score calculated using multiple socio-demographic and economic factors, including financial resources of the residents, housing conditions, motorisation level, education and employment profile [24]. The SES ranks are calculated periodically by the ICBS using internationally accepted methods including factor analysis and the Ward's methods [25] to determine the SES ranks for each geographic area. The SES rank of residential areas was correlated with Gini index [26], a measure of income inequalities (Spearman's *r* coefficient 0•71 *P*<0.001).

Population group was determined based on the ICBS classification of the predominant population (Arab or Jewish) in each city/town. The few cities with mixed Arab and Jewish residents (such as Haifa and Jerusalem) were classified as Jewish cities, as Jews comprised the majority of the population (>60%). Classification of ultra-Orthodox Jewish towns relied on voting patterns for the ultra-Orthodox and religious parties in the Knesset (the Israeli parliament). The variable population group was considered as a social determinant of COVID-19, given prior evidence linking between this variable and infectious disease incidence and vaccine uptake [15,17] in Israel.

### 3.4 Data analysis

Overall, age and sex specific cumulative incidence (per 1000 persons) of SARS-CoV-2 infection and mortality rates (per 100,000) were calculated using the corresponding numbers of new PCR-confirmed SARS-CoV-2 cases and COVID-19 deaths, respectively, in the numerators and population size [9] in the denominators. Age-standardised incidence and mortality rates (and standard errors) were calculated separately for males and females using the direct standardisation method in which the weights were determined as the age distribution of the total Israeli population in 2019. To examine differences in the incidence rates of SARS-CoV-2 infection and COVID-19 mortality amongst age groups, we calculated the relative risk (RR) using the youngest age group as the reference (comparison group). We also calculated the difference in the infection incidence and mortality between males and females; in these calculations, females were the reference (comparison group). Confidence intervals (95% CI) for the RR in these analyses were calculated using the method described by Armitage and Berry [27]. The prevalence proportion of hospitalised COVID-19 patients was calculated as the number of hospitalised patients on each day divided by the average population size (expressed per 100,000 persons). Uptake of the COVID-19 vaccine was calculated using the cumulative number of immunised persons in the numerators and population size in the denominators; this was done for the first and second vaccine doses, overall and by age groups.

To examine differences between residential SES and population group in SARS-CoV-2 infection incidence rates, diagnostic tests uptake, COVID-19 vaccine uptake and COVID-19 mortality, general linear models with negative binomial distribution and log function were performed separately for each of these variables. In each model, the dependant variable was count data (e.g., the number of PCR-confirmed cases), the variables of residential SES and population group were the independent variables and the logarithmic transformation of the population size in each town was included as an offset. The RR and their corresponding 95% CI and P values were obtained from these models. This analysis was limited to 260 towns with 2000 residents or more to obtain robust estimates. Towns with fewer than 15 confirmed cases or deaths were excluded from the analysis since the exact number of cases was lacking. In the analysis of mortality, we included in the model the variable median age of each town, which was available for 194/260 (75%) of the towns. Data were analysed using SPSS version 27 (IBM Corp, Armonk, NY), R version 4.0.3 and WINPEPI software.

### 3.5 Ethics

We analysed publicly available aggregated data. The Ethics Committee of Tel Aviv University approved the study.

### 3.6 Role of the funding source

There was no external funding for this study.

## 4 Results

Between March 15, 2020 and February 27, 2021, 774,030 persons overall had PCR-confirmed SARS-CoV-2 infection. The cumulative incidence was 84•5 per 1000 persons. Information on age or sex was missing for 8786 (1•1%) persons. The overall incidence rates were similar amongst males and females. The highest incidence rates were found in young adults aged 20–29 years. Incidence rates decreased but remained stable between the ages of 30–59 years and further declined amongst the older ages. Incidence rates were slightly higher in males vs. females after the age of 60 (Table 1).

**Table 1:** The cumulative incidence rates of PCR-confirmed SARS-CoV-2 infection by age and sex, Israel March 15, 2020 – February 27, 2021.

	Overall			Males			Females			
Age group, years	Number of cases	Incidence rate per 1000	Relative risk (95% CI) Age groups	Number of cases	Incidence rate per 1000	Relative risk (95% CI) Age groups	Number of cases	Incidence rate per 1000	Relative risk (95% CI) Age groups	Relative risk (95% CI) Males vs. females
Overall	774,030	84•‏5	–	381,481	84•8*^§^	–	383,763	84•1*^§^	–	0•99 (0•98–1•00)
0–19	268,279	82•1	1•00 (Ref.)	140,350	83•8	1•00 (Ref.)	127,929	80•4	1•00 (Ref.)	1•04 (1•03–1•05)
20–24	81,960	125•9	1•53 (1•52–1•54)	41,194	124•2	1•48 (1•47–1•50)	40,766	127•7	1•59 (1•57–1•61)	0•97 (0•96–0•98)
25–29	65,006	105•3	1•28 (1•27–1•29)	31,163	99•8	1•19 (1•18–1•20)	33,843	111•0	1•38 (1•36–1•40)	0•90 (0•89–0•91)
30–34	56,453	94•3	1•15 (1•14–1•16)	27,601	92•0	1•10 (1•08–1•11)	28,852	96•5	1•20 (1•19–1•21)	0•95 (0•94–0•97)
35–39	50,810	87•4	1•06 (1•05–1•07)	24,546	84•9	1•01 (1•00–1•03)	26,264	89•9	1•12 (1•10–1•13)	0•94 (0•93–0•96)
40–44	47,237	84•3	1•03 (1•02–1•04)	22,277	80•3	0•96 (0•94–0•97)	24,960	88•4	1•10 (1•08–1•11)	0•91 (0•89–0•92)
45–49	44,726	87•8	1•07 (1•06–1•08)	21,419	85•1	1•02 (1•00–1•03)	23,307	90•5	1•13 (1•11–1•14)	0•94 (0•92–0•96)
50–54	37,183	86•8	1•06 (1•05–1•07)	17,774	84•3	1•01 (0•99–1•02)	19,409	89•2	1•11 (1•09–1•13)	0•94 (0•93–0•96)
55–59	31,065	78•4	0•95 (0•94–0•96)	15,122	79•0	0•94 (0•93–0•96)	15,943	77•8	0•97 (0•95–0•98)	1•01 (0•99–1•04)
60–64	25,010	66•7	0•81 (0•80–0•82)	12,595	70•7	0•84 (0•83–0•86)	12,415	63•0	0•78 (0•77–0•80)	1•12 (1•10–1•15)
65–69	19,452	54•8	0•67 (0•66–0•68)	10,014	60•1	0•72 (0•70–0•73)	9438	50•1	0•62 (0•61–0•64)	1•20 (1•17–1•23)
70–74	13,860	48•5	0•59 (0•58–0•60)	7075	53•7	0•64 (0•63–0•66)	6785	44•0	0•55 (0•53–0•56)	1•22 (1•18–1•26)
75–79	8458	51•3	0•62 (0•61–0•64)	4103	56•2	0•67 (0•65–0•69)	4355	47•4	0•59 (0•57–0•61)	1•18 (1•14–1•23)
≥80	15,745	58•3	0•71 (0•70–0•72)	6248	58•0	0•69 (0•68–0•71)	9497	58•5	0•73 (0•71–0•74)	0•99 (0•96–1•02)

CI: confidence intervals; PCR: polymerase chain reaction; Ref: reference.

Information on age or sex was missing for 8786 (1•1%) persons.

⁎ Crude incidence rates. ^§^Age-standardized (adjusted) rates were calculated using the age distribution of the total Israeli population as weights and they were similar: 84•4 per 1000 (standard error 0•13) in males and 84•6 per 1000 (standard error 0•13) in females.

Overall, 5687 COVID-19 patients had died, yielding a mortality rate of 62•8 per 100,000 persons. The mortality rates increased substantially with age and were higher amongst males than females (Table 2).

**Table 2:** COVID-19 mortality rates per 100,000 by age and sex groups (*N* = 5687) up to February 27, 2021.

	Overall			Males			Females			
Age, years	Number of cases	Mortality rate per 100,000	Relative risk (95% CI) Age groups	Number of cases	Mortality rate per 100,000	Relative risk (95% CI) Age groups	Number of cases	Mortality rate per 100,000	Relative risk (95% CI) Age groups	Relative risk (95% CI) Males vs. females
All ages	5687	62•8	–	3263	72•6*^,^§	–	2424	53•1*^,^§	–	1•37 (1•30–1•44) *^,^§
<65	765	9•6	Reference	522	13•0	Reference	243	6•1	Reference	2•12 (1•82–2•47)
65–74	1176	183•7	19•16 (17•49–20•98)	787	264•1	20•32 (18•19–22•69)	389	113•6	18•54 (15•79–21•76)	2•32 (2•06–2•62)
75–84	1658	543•3	56•66 (52•01–61•73)	1005	762•5	58•66 (52•78–65•19)	653	376•6	61•43 (53•02–71•17)	2•02 (1•84–2•23)
≥85	2088	1614•8	168•43 (155•07–182•94)	949	1944•7	149•59 (134•49–166•39)	1139	1414•9	230•79 (200•97–265•02)	1•37 (1•26–1•49)

CI: confidence intervals.

⁎ Crude morality rate.

§ Age-standardized (adjusted) mortality rate using the age distribution of the total Israeli population as weights were: 83•3 per 100,000 (standard error 1•4) in males and 46•1 per 100,000 (standard error 0•9) in females: comparing the age-adjusted mortality rates between males vs. females yielded a relative risk of 1•81 (95% CI 1•71–1•91).

**Fig. 1:**
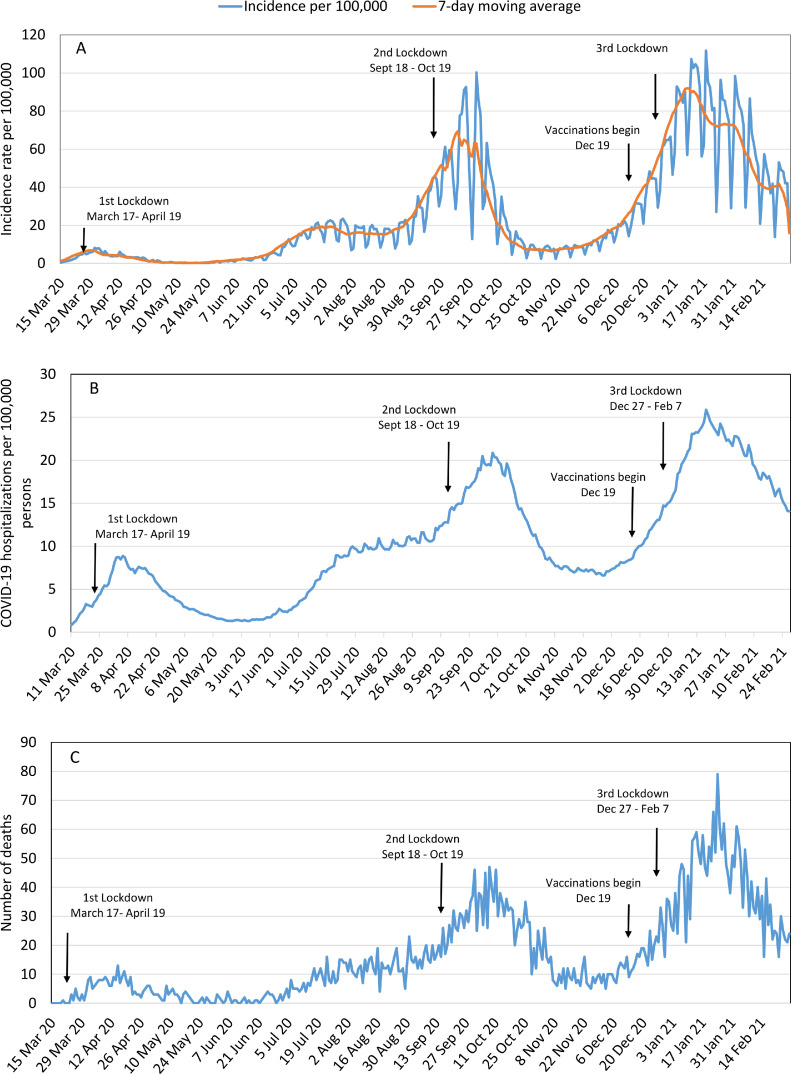
Epidemiological curve of SARS-CoV-2 infection in Israel, March 2020–February 2021. COVID-19: Coronavirus disease 2019; SARS-CoV-2: Severe acute respiratory syndrome coronavirus 2. (A) Daily incidence rate (per 100,000 persons) of PCR-confirmed SARS-CoV-2 infection (blue line) and 7-day moving average (orange line); (B) Prevalence proportion of COVID-19 hospitalisations (per 100,000 persons); (C) Daily number of COVID-19 deaths.

The epidemiological curves demonstrate three waves of SARS-CoV-2 infection in Israel; each resulted in the implementation of lockdown, which typically was followed by a reduction in the COVID-19 incidence, hospitalisations and mortality (Fig. 1). A consistent decrease was observed in COVID-19 incidence and mortality since mid-January 2021, one month after the introduction of COVID-19 vaccination. The epidemiological curves were similar in all age groups **(Supplementary Figure 2).**

### Population subgroups and residential SES

**Fig. 2:**
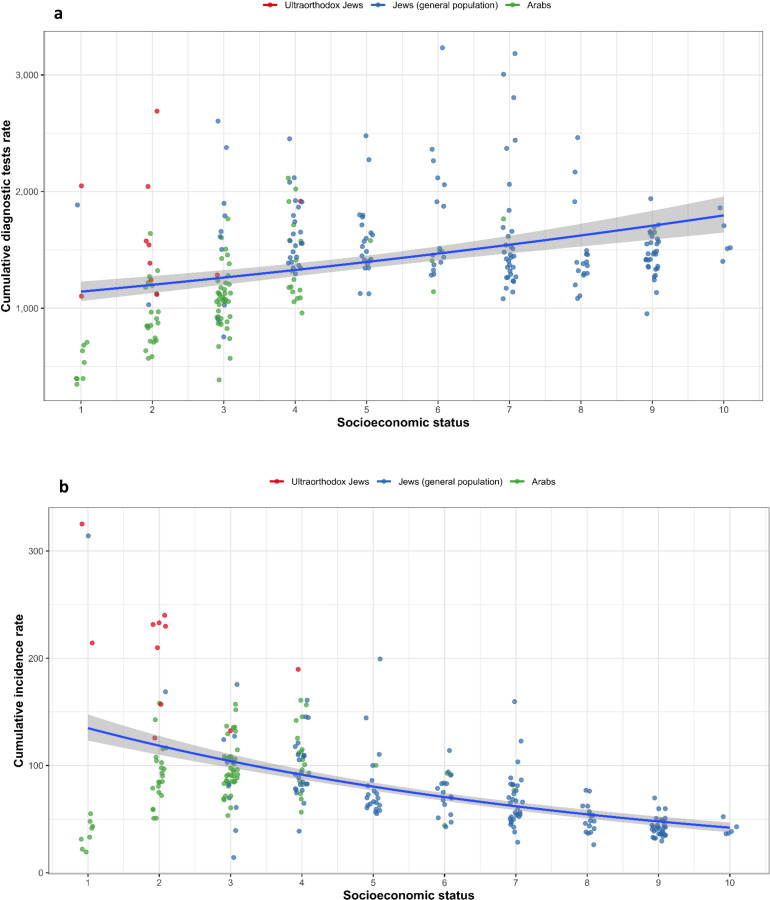
Uptake of SARS-CoV-2 PCR test (per 1000 persons), SARS-CoV-2 incidence rates (per 1000 persons) according to subpopulation group and residential SES. PCR: Polymerase chain reaction; SARS-CoV-2: Severe acute respiratory syndrome coronavirus 2; SES: socioeconomic status. (a) Cumulative uptake rate (per 1000 persons) of diagnostic SARS-CoV-2 PCR test (b) Cumulative rate of PCR-confirmed SARS-CoV-2 infection in 260 towns with 2000 residents or more, according to residential SES ranks (10 being the highest SES and 1 being the lowest SES), and population subgroup. Each circle represents a town; red circles=towns with mostly ultra-Orthodox Jewish residents, green circles=Arab towns and blue=towns with mostly general Jewish populations, and trends line in relation to SES rank.

The uptake of the SARS-CoV-2 PCR test was lower in towns with predominantly Arab residents than in towns with a mainly general Jewish population (adjusted RR 0•75 [95% CI 0•68–0•82]), but a higher uptake was found in towns with a predominantly ultra-Orthodox Jewish population (adjusted RR 1•35 [95% CI 1•12–1•63]). Compared to the towns with the lowest SES, the uptake of the test increased 1.4–2.0-fold in towns of higher SES (Fig. 2A, Table 3).

**Table 3:** General linear models of the associations of residential SES and predominant population group with the uptake of SARS-CoV-2 PCR testing, cumulative incidence of SARS-CoV-2 infection and COVID-19 mortality, Israel.

	PCR diagnostic tests ^a^			Cumulative SARS-CoV-2 infection incidence ^b^			Cumulative COVID-19 mortality ^c^		
Variable	RR	95% CI	p-value	RR	95% CI	p-value	RR	95% CI	p-value
**Residential SES rank**									
**SES**-1	1•00			NA			NA		
**SES**-2	1•41	1•18–1•68	<0•001	1•00			1•00	–	–
**SES**-3	1•66	1•40–1•96	<0•001	1•01	0•87–1•16	0•90	0•80	0•58–1•12	0•20
**SES**-4	1•96	1•64–2•34	<0•001	1•08	0•93–1•27	0•30	0•81	0•49–1•31	0•40
**SES**-5	1•82	1•49–2•23	<0•001	0•89	0•73–1•08	0•20	0•54	0•29–1•01	0•049
**SES**-6	2•01	1•64–2•46	<0•001	0•79	0•65–0•97	0•022	0•54	0•29–1•02	0•054
**SES**-7	1•87	1•54–2•26	<0•001	0•74	0•61–0•89	0•001	0•47	0•26–0•84	0•011
**SES**-8	1•67	1•35–2•06	<0•001	0•55	0•45–0•68	<0•001	0•38	0•20–0•72	0•003
**SES**-9	1•66	1•36–2•01	<0•001	0•46	0•38–0•56	<0•001	0•31	0•16–0•57	<0•001
**SES**-10	1•81	1•37–2•41	<0•001	0•45	0•33–0•62	<0•001	NA		
**Population group**									
Jewish general population	1•00	–	–	1•00	–	–	1•00	–	–
Ultraorthodox Jews	1•35	1•12–1•63	<0•001	2•10	1•66–2•67	<0•001	1•21	0•76–1•93	0•40
Arabs	0•75	0•68–0•82	<0•001	1•05	0•94–1•18	0•40	1•49	1•06–2•10	0•018

CI: Confidence intervals; NA: not applicable; PCR: Polymerase chain reaction; RR: Relative risk; SARS-CoV-2: severe acute respiratory syndrome coronavirus 2; SES: Socioeconomic status (10 is the highest SES and 1 is the lowest).

Multivariable general linear models were conducted with negative binomial distribution and log function using data of towns with more than 2000 residents, cumulative counts of each outcome were included as the dependant variable in three separate models, and the natural logarithm of the population size included as an offset. Each model included residential SES rank and population group. ^a^ The analysis of PCR uptake was based on data of 260 towns. **^b^** The analysis of incidence was based on data of 249 towns since we excluded the 11 towns with the lowest SES rank (outlier observations). **^c^** The analysis of mortality was based on data of 65 towns with 15 or more COVID-19 deaths that have information on median age (which was included in the model as covariate: adjusted RR 1•06 95% CI 1•03–1•09) *p*<0•001). Towns with the lowest SES rank were excluded from this analysis (outlier observations). No deaths were reported in towns with residential SES of 10 (highest).

**Fig. 3:**
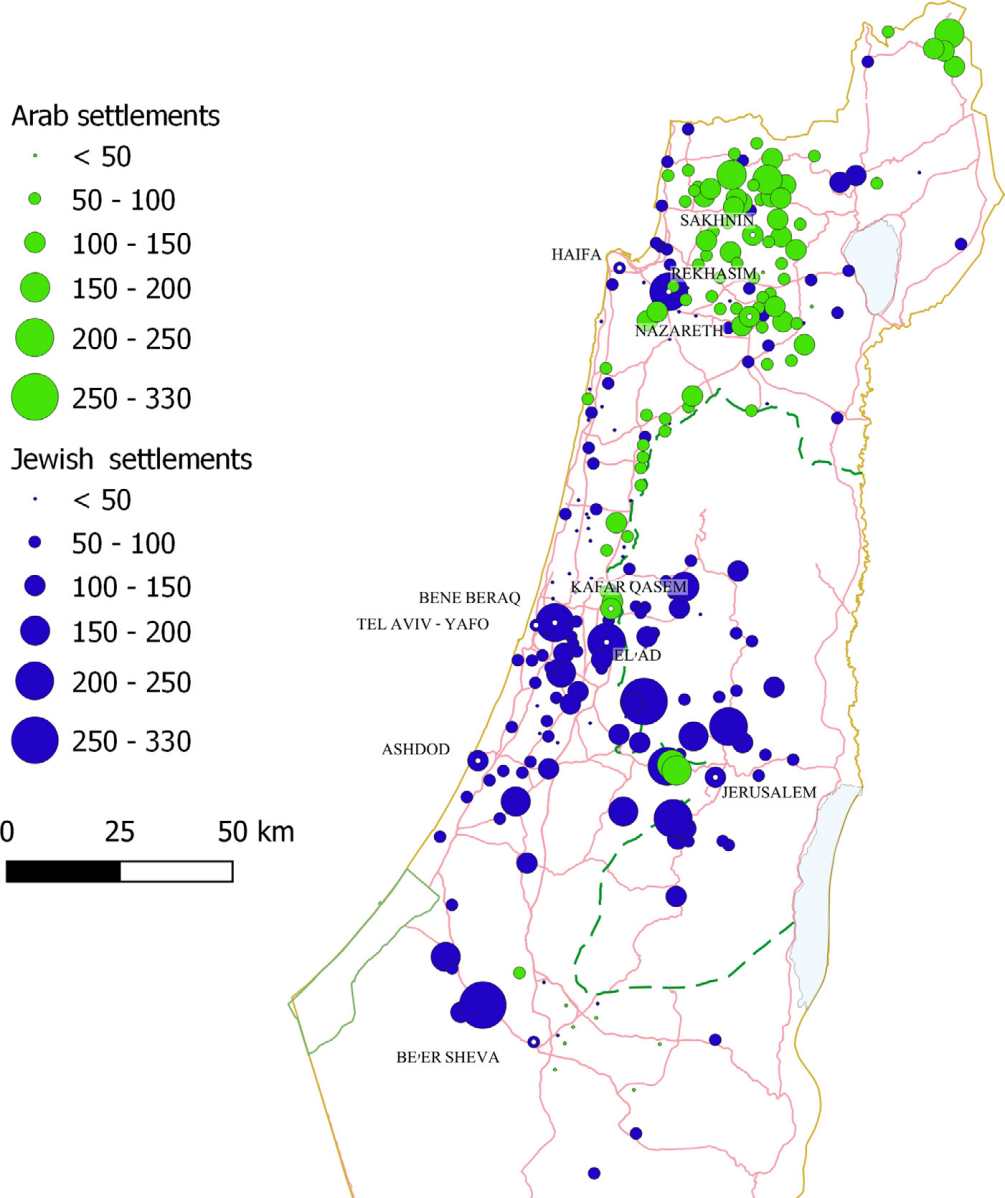
Cumulative incidence rates (per 1000 persons) in towns with 2000 residents or more according to population group and geographic distribution. Towns with mixed Arab and Jewish residents were classified as towns with mostly Jewish population since more than 60% of the population was Jewish. Each circle represents a town; the larger the circle, the higher the incidence rate. Circles in green represent towns with predominantly Arab population (e.g., Nazareth, Sakhnin, Kafar Qasem), Circles in blue represent towns with predominantly Jewish populations; the towns Rekhasim, Bene Beraq (Bnei Brak) and Elad represent towns with mainly ultra-Orthodox Jewish populations.

The incidence rates of SARS-CoV-2 infection were lowest in Arab towns with the lowest SES rank, mainly Bedouin towns in the south in which SARS-CoV-2 testing was also the lowest. We therefore excluded towns with the lowest SES rank from the incidence and mortality regression analysis. The risk of SARS-CoV-2 infection was higher in towns with a predominantly Arab population (RR 1•42 [95% CI 1•28–1•57]) or an ultra-Orthodox Jewish population (RR 2•83 [95% CI 1 2•22–3•67]) compared to the general Jewish population. In an SES-adjusted model, the difference between the ultra-Orthodox and general Jewish sub-populations remained significant. This model showed lower risk for SARS-CoV-2 infection in higher SES towns which include a mainly general Jewish population (Fig. 2B, Table 3). The geographic distribution of the towns and the incidence rates of SARS-CoV-2 infection is presented in Fig. 3.

The risk of COVID-19 mortality was higher in Arab-towns compared to towns with a mainly general Jewish population after adjustment for median age of each town and residential SES (adjusted RR 1•49 [95% CI 1•06–2•10]) (Table 3).

### 4.1 Non-pharmaceutical prevention and control measures

**Fig. 4:**
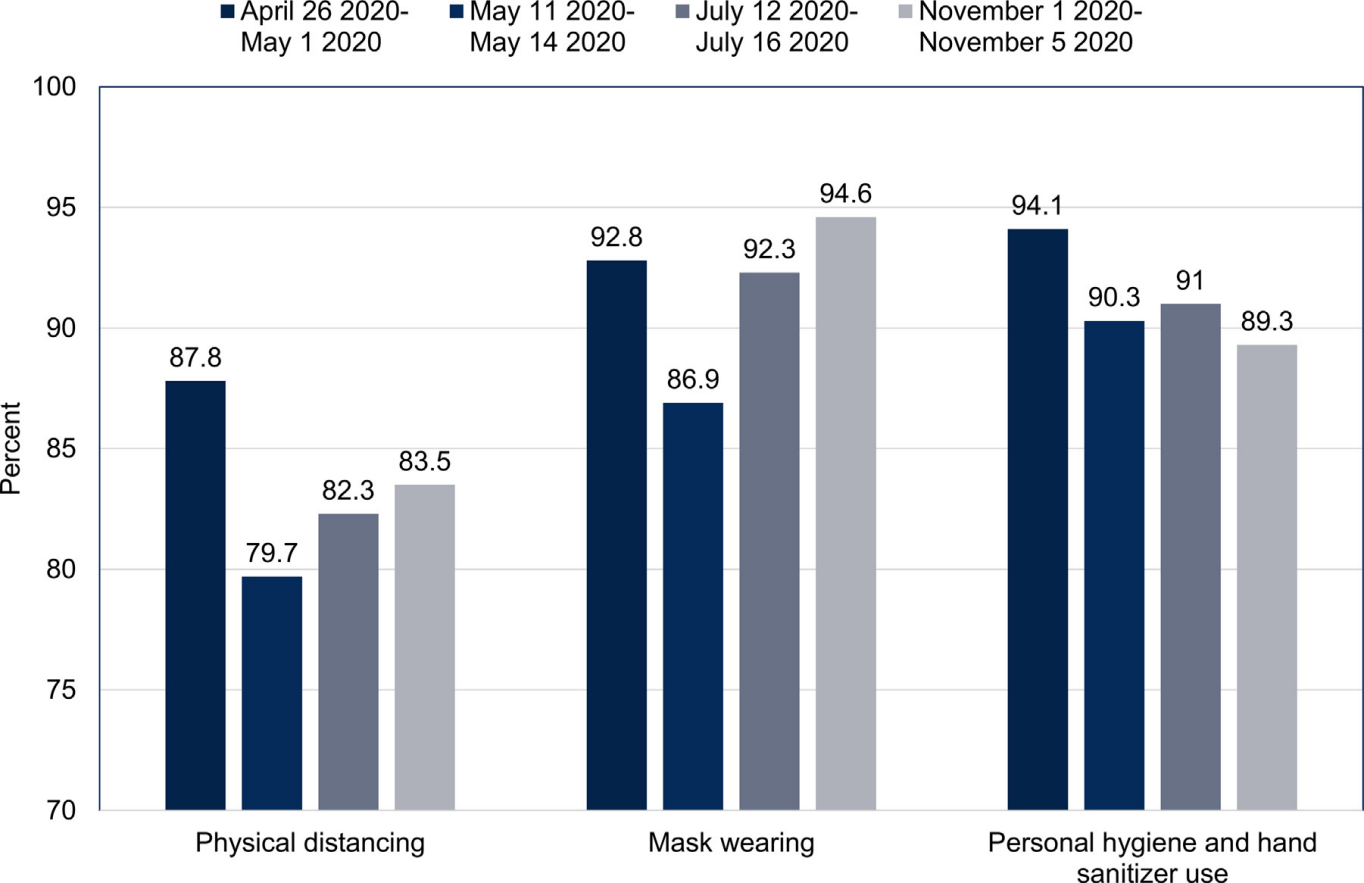
Adherance to COVID-19 non-pharmaceutical preventive measures amongst adults, Israel 2020. Results from periodical national surveys conducted by the Israel Central Bureau of Statistics on public adherence to COVID-19 non-pharmaceutical preventive measures. Adherence was defined based on the self-reports of the participants in these surveys to the question of to which degree they complied with the recommendation of maintaining two-metre physical distancing between people in public settings, wearing a mask, and personal hygiene such as hand washing and use of hand sanitiser.

Self-reported adherence to physical distancing, wearing face masks and personal hygiene practices was highest in the early phase of the epidemic, weakened after the first lockdown, but increased in later surveys (Fig. 4).

### 4.2 COVID-19 vaccination

**Fig. 5:**
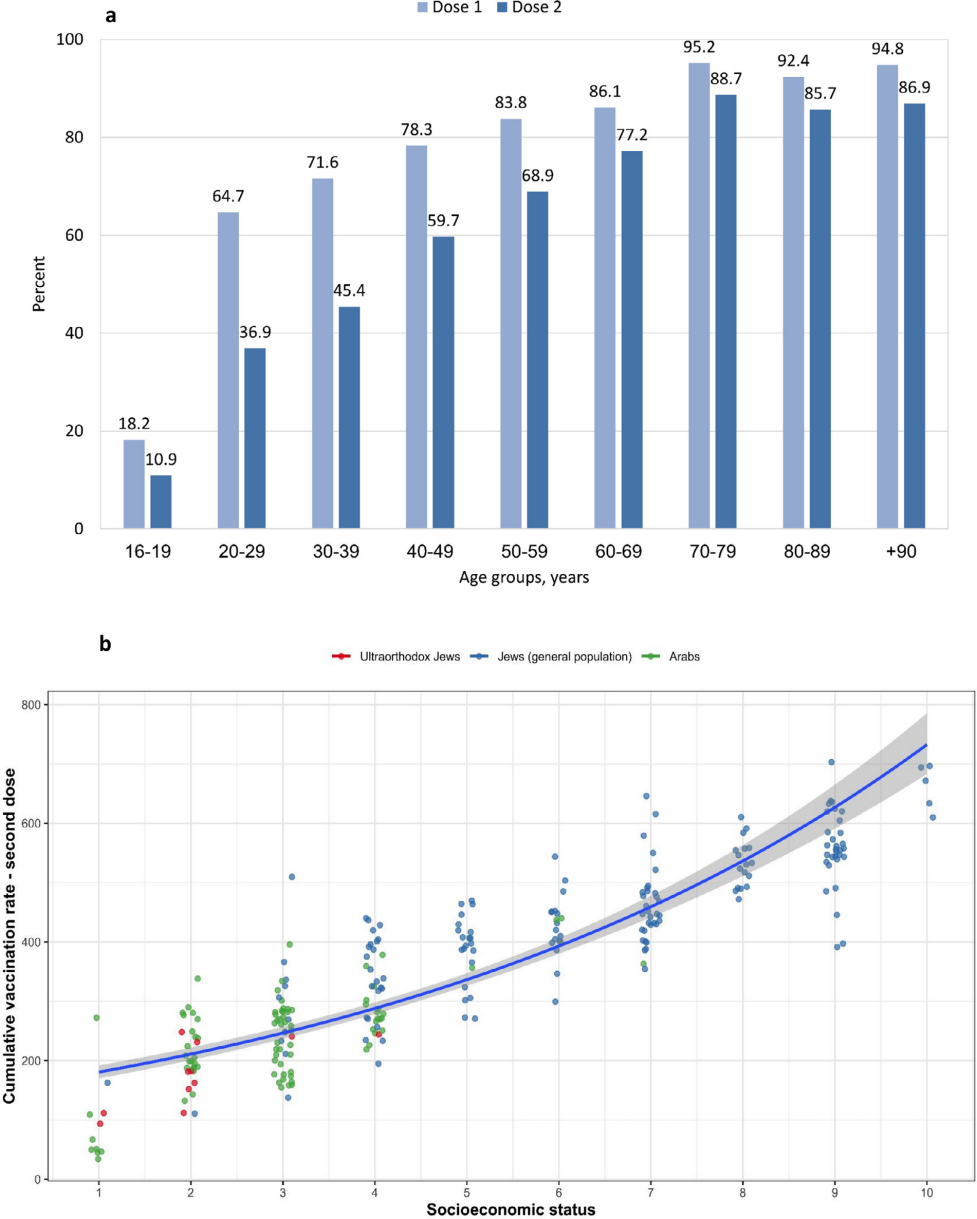
BNT162b2 COVID-19 vaccine uptake by age and vaccine dose (a), population subgroup and residential SES (b). COVID-19: Coronavirus disease 2019; SES: socioeconomic status. (a): The percentage of vaccinated people in each age group, dose 1 in light blue and dose 2 in dark blue. (b): Cumulative uptake rate (per 1000 persons) of the second dose of COVID-19 vaccine in all age groups in each town. An analysis of 260 towns with 2000 residents or more, according to residential SES ranks (10 being the highest SES and 1 being the lowest SES), and population subgroup. Each circle represents a town; red circles=towns with mostly ultra-Orthodox Jewish residents, green circles=Arab towns and blue=towns with mostly general Jewish populations, and trends line in relation to SES rank.

As of March 2, 2021, 4•82 million people were immunised with the first vaccine dose and 3•51 million received the second dose, representing a coverage of 53•2% and 38•8%, respectively, in the general population in Israel. The vaccine coverage increased with age and exceeded 80% in individuals aged 50 years or older for the first dose and 69% for the second dose. Vaccine uptake was lower in towns with mainly Arab and ultra-Orthodox Jewish populations compared to the general Jewish population, and it increased with improved residential SES (Fig. 5 and Table 4).

**Table 4:** General linear model of the association of residential SES and predominant population group with the uptake of the COVID-19 vaccine (second dose), Israel.

Variable	RR	95% CI	p-value
**Residential SES rank**			
**SES**-1	1•00		
**SES**-2	2•29	1•96–2•67	<0•001
**SES**-3	2•60	2•24–3•01	<0•001
**SES**-4	3•06	2•62–3•57	<0•001
**SES**-5	3•52	2•95–4•22	<0•001
**SES**-6	3•98	3•33–4•77	<0•001
**SES**-7	4•18	3•52–4•97	<0•001
**SES**-8	4•83	4•00–5•83	<0•001
**SES**-9	5•05	4•25–6•02	<0•001
**SES**-10	6•00	4•68–7•69	<0•001
**Population group**			
Jewish general population	1•00	–	–
Ultraorthodox Jews	0•77	0•65–0•91	0•002
Arabs	0•85	0•77–0•92	<0•001

CI: Confidence intervals; RR: Relative risk; SES: Socioeconomic status (10 is the highest SES and 1 is the lowest).

Multivariable general linear model was conducted with negative binomial distribution and log function using data of towns with more than 2000 residents, cumulative count of vaccinated people with the second dose of BNT162b2 COVID-19 vaccine was included as the dependant variable, and the natural logarithm of the population size included as an offset. The model included residential SES rank and population group.

## 5 Discussion

The SARS-CoV-2 infection has affected all ages, both sexes and various subpopulations and SES strata in Israel. However, variation in the infection incidence and mortality rates was found across these sociodemographic strata. The highest incidence rate of SARS-CoV-2 was found in young adults aged 20–29 years, while the lowest incidence rate was found in individuals aged 60 years and older. This is likely due to differences between the age groups in risk perception and adherence to risk mitigation measures [28]; young people commonly have a low personal risk perception of COVID-19 and thus tend to adhere less to preventive measures [28], [29], [30].

COVID-19 mortality rates increased amongst the elderly, which is in agreement with previous reports [31], likely due to the high burden of comorbidities and more severe disease in this group [32]. Interestingly, COVID-19 mortality affected men disproportionately compared to women. Similar findings were reported in other countries [31]. Men also experience more severe disease than women [33], which might be explained by higher inflammatory response involving the innate immunity in men [33].

Importantly, the infection incidence rates was interrupted with the implementation of non-pharmaceutical preventive measures such as school closures, quarantine and wearing face masks in public and lockdowns. While quarantine has been successfully implemented historically in the containment of epidemics, evidence on the effectiveness of wearing face masks by the general public in stopping the virus transmission in early 2020 was initially elusive. Since then, compelling evidence has accumulated indicating that adequate face mask wearing is associated with significant protection against SARS-CoV-2 infection, as well as physical distancing [34,35].

Remarkably, the year 2020 as a whole was characterised by lower rates of infectious respiratory illnesses than the preceding years in Israel [36]. Possible explanations might be changes in public behaviours and implementation of the aforementioned non-pharmaceutical preventive measures, effectively impacting other infectious agents, and thus reducing visits to physicians.

One might expect that since SARS-CoV-2 is a novel virus that evaded the naïve human populations, it would equally affect all sociodemographic strata. However, we observed substantial social disparities related to the SARS-CoV-2 infection rates according to SES and population groups. Specifically, we found higher risk of infection amongst Arab and ultra-Orthodox communities that typically display lower SES than the general Jewish population. The utilisation of PCR testing for SARS-CoV-2 was lower in the Arab population. These findings are alarming given that PCR testing is free-of-charge and the enormous effort was invested in enhancing accessibility to testing, joint work with local community leaders and advertising in various languages.

The low uptake of PCR testing could be the result of low awareness of the severity of COVID-19 or concerns about being put in quarantine if tested positive, which has a huge economic impact on these families. It was shown that persons would adhere to quarantine guidelines if given financial compensation [37]. Moreover, home quarantine is not always feasible for individuals in Arab and ultra-Orthodox Jewish communities due to high household crowding that likely enhances household transmission of SARS-CoV-2. Furthermore, quarantine in coronavirus hotels subsidised by the government is not always considered culturally acceptable by these populations. Violation of the control measures forbidding large gatherings such as weddings and funerals were reported in both the Arab and ultra-Orthodox Jewish populations. Taken all together, these factors likely explain the differential incidence of the infection by sub-population group.

Importantly, we also found a positive association between residential SES and PCR testing, irrespective of population group, and a negative association between residential SES and incidence of the SARS-CoV-2 infection. Access to healthcare in Israel is universal; nonetheless, there was a higher COVID-19 mortality risk amongst Arabs compared to the general Jewish population. Our findings agree with emerging evidence demonstrating social disparities in COVD-19. For example, studies from the United States showed that racial/ethnic minority status, SES and household characteristics were related to COVID-19 incidence and mortality rates [4,6]. Supportive findings were reported in the United Kingdom [5,38]. A study from Sweden showed that migrants from Middle-Eastern countries, Africa and other Nordic countries had a higher COVID-19 mortality risk compared to Swedish-born individuals [39]. The first step towards reducing such disparities is quantifying and understanding the roots of these disparities [40] and closing gaps in social disparities in general; the allocation of more resources during the epidemic should therefore take into account any pre-existing inequalities. Minority groups might be more vulnerable to misinformation regarding COVID-19 [41]. Therefore, interventions to reduce social disparities during the COVID-19 epidemic should be enhanced amongst minorities and low SES communities, including educational programs in the native population's language. Enhancing access to immunisation (e.g., more immunisation centres in peripheral regions and populations, longer working hours in immunisation centres, immunisation at workplaces) can increase the vaccine uptake. Improving trust in policy makers is essential to achieving compliance [42], therefore working with local community leaders should be given a high priority and the inclusion of professional representatives of minority groups should be an integral part of decision-making [38].

Vaccines are the most equitable preventive measure. At the national level, COVID-19 immunisation with BNT162b2 vaccine has been successful considering the complex logistics needed for the adequate handling and storage of this vaccine. Despite this success, the vaccine uptake in Arab and ultra-Orthodox Jewish communities has been shown to be lower compared to the general Jewish population. Moreover, the vaccine uptake was weaker in low SES areas compared to higher residential SES areas.

Based on earlier evidence suggesting R_0_ between 2 and 3 for SARS-CoV-2 [43], the herd immunity level was estimated at 60–70% to halt/reduce the virus transmission. The current success of COVID-19 immunisation, and the high real-world vaccine effectiveness [44], the Israeli population likely is approaching the lower early estimate of herd immunity level. One month after starting COVID-19 immunisation and increased coverage, a consistent decrease was found in the disease incidence and mortality, especially amongst the older age groups. Although this early evidence is highly encouraging, new challenges are imposed with the emergence of the B.1.1.7 (UK) variant which is highly transmissible and causes more severe disease than previously known variants [45]. Reduced neutralising antibody responses to the B.1.1.7 lineage following vaccination with BNT162b2 vaccine has been demonstrated [46]. A real-world study from Israel showed high BNT162b2 vaccine effectiveness [44] during a period when the UK variant was circulating in Israel, thus we anticipate that the vaccine will provide protection, although the duration of protection has yet to be determined and new vaccine generation might be needed. The B.1.351 (South Africa) variant is rare in Israel (MOH telegram). Accordingly, expanding and enhancing molecular surveillance of SARS-CoV-2 is warranted to assess the emergence of new variants, and their impact on population immunity.

Our study has strengths. The case-definition of SARS-CoV-2 infection relied on PCR testing. We included data of various populations and SES levels, making our findings generalisable to other countries. As access to healthcare is universal in Israel, our study controlled for the financial barriers to healthcare.

Our study also has limitations: 1) there might be self-selection for SARS-CoV-2 testing; 2) no worthwhile information was available on the indications for testing (e.g., symptoms, asymptomatic contacts); and 3) the classification of population group was based on the predominant population that lived in each town, which might result in some misclassification, for example, a few mixed cities that have both Arab and Jewish residents (e.g., Haifa, Tel Aviv-Jaffa) were classified as predominately Jewish cities since Jews were more than 60% of the population. Our study also lacked individual-level SES data. Missing information on age and sex was low (1•1%). Exact information on the number of deaths was lacking in towns with fewer than 15 COVID-19 fatalities, so the analysis of COVID-19 deaths by population group and residential town relied on partial information.

In conclusion, our study provides new evidence that links social determinants with COVID-19 burden and vaccine uptake, with minority groups and low SES communities experiencing the highest burden and lowest vaccine uptake. Remarkably, these inequalities were evident despite high accessibility to healthcare and COVID-19 vaccines in Israel. The recognition of the COVID-19 disparities as demonstrated herein should stimulate future in-depth studies to understand the causes of COVID-19 social inequalities. Interventions to reduce COVID-19 disparities should be enhanced amongst minorities and low SES communities to adequately control the pandemic and to better mitigate the health and social consequences.

## Contributors

KM, MSG and DC conceived and designed the study. KM, GH, WN, YL, SG and YA undertook the analysis and took responsibility for the integrity of the data and the accuracy of the data analysis. All authors had full access to all of the data in the study. KM, MSG and DC drafted the manuscript. All authors critically revised the manuscript for important intellectual content and gave final approval for the version to be published. All authors agree to be accountable for all aspects of the work in ensuring that questions related to the accuracy or integrity of any part of the work are appropriately investigated and resolved.

## Declaration of interests

All authors declare no conflict of interest.

## Data availability statement

Data used in the current analysis on SARS-CoV-2 cases, COVID-19 hospitalisations and deaths as well as on COVID-19 immunisation are publicly available [23] (https://data.gov.il/dataset/covid-19).

Data on the population size and residential socioeconomic status and population compositions are available through the Israel Central Bureau of Statistics website and publications.

https://www.cbs.gov.il/en/subjects/Pages/Demographic-Characteristics.aspx

https://www.cbs.gov.il/en/publications/Pages/2019/Characterization-and-Classification-of-Geographical-Units-by-the-Socio-Economic-Level-of-the-Population-2015.aspx

## Appendix Supplementary materials

Image, application 1
