# Supplementary material for: A nationwide analysis of population group differences in the COVID-19 epidemic in Israel, February 2020–February 2021
Source: Lancet Reg Health Eur. 2021 Jun 5;7:100130. doi: 10.1016/j.lanepe.2021.100130 (PMC8177966; doi:10.1016/j.lanepe.2021.100130)
Supplement: Supplementary file 1 [file mmc1.pdf]

Supplementary material

Article title

A nationwide analysis of population group differences in the COVID-19 epidemic in Israel, February 2020–February 2021

Khitam Muhsen PhD,[1]* Wasef Na'aminh PhD,[1] Yelena Lapidot PhD,[1] Sophy Goren BSc[1], Yonatan Amir BSc,[1] Saritte Perlman, MPH,[1] Manfred S. Green MBChB,[2] Gabriel Chodick PhD,[1,3] Dani Cohen PhD[1]

[1] Department of Epidemiology and Preventive Medicine, School of Public Health, Sackler Faculty of Medicine, Tel Aviv University, Tel Aviv, 69978

Israel

[2] University of Haifa, School of Public Health, Haifa, Israel

[3] Maccabi Institute for Research & Innovation, Maccabi Healthcare Services, Kaufman 4, Tel Aviv, Israel

Supplementary figure 1: Number of daily diagnostic PCR tests for the detection of SARS-Cov-2 in Israel, February 2020- February 2021 (N= 10,190,185)

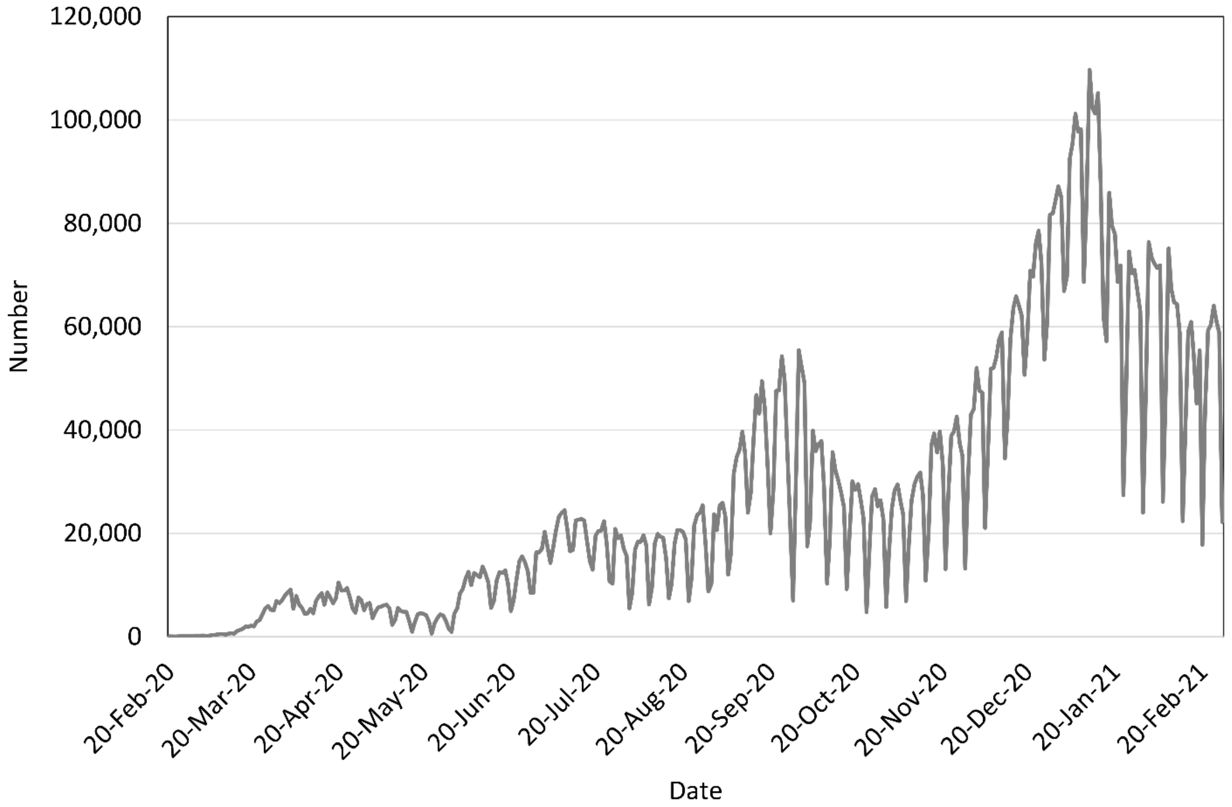

SARS-CoV-2 – severe acute respiratory syndrome coronavirus 2; PCR – Polymerase chain reaction

Supplementary figure 2: Incidence rates (per 100,000) of PCR-confirmed SARS-CoV-2 infections by sex and age groups, Israel, March 15, 2020-February 27, 2021

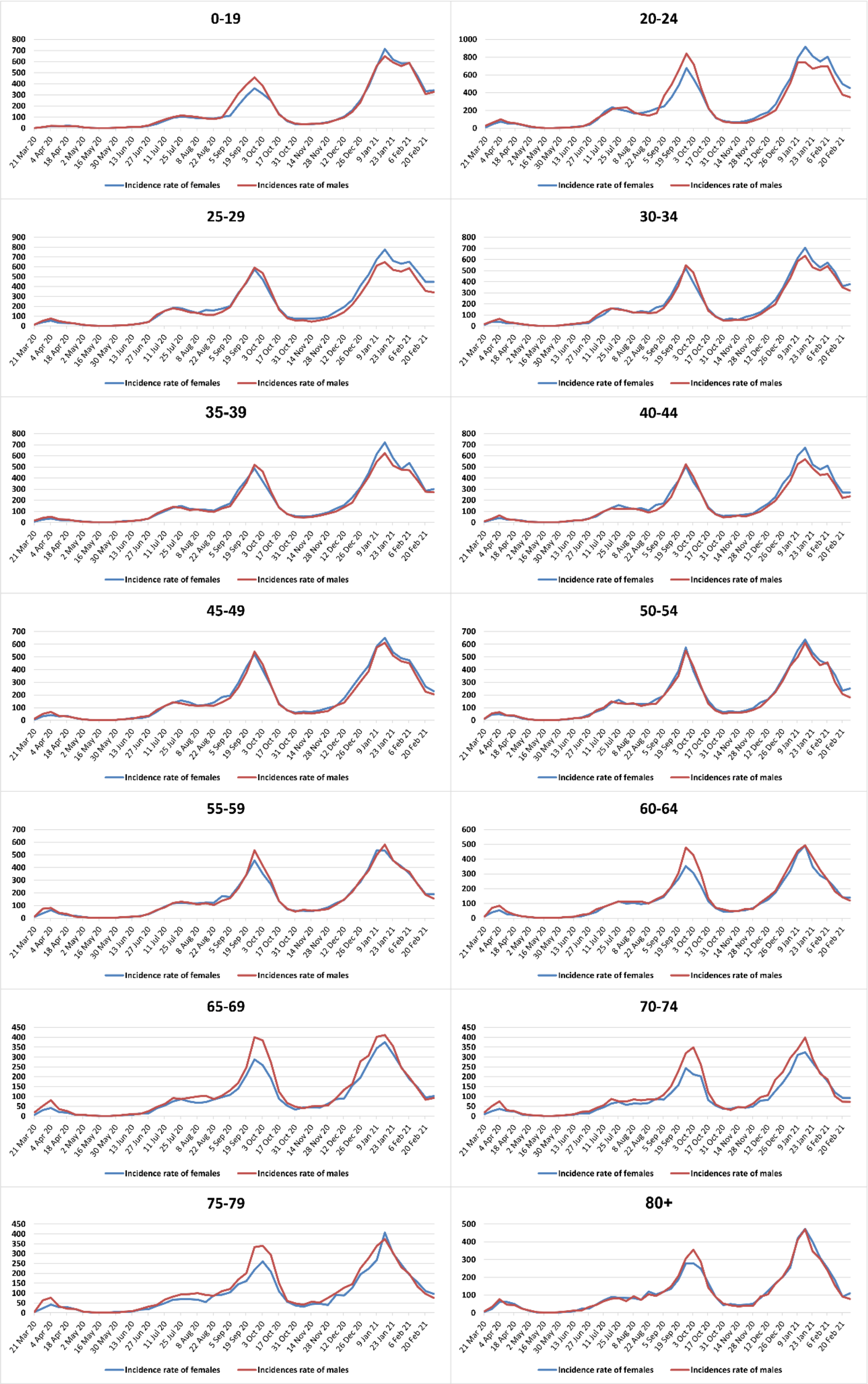

SARS-CoV-2 – severe acute respiratory syndrome coronavirus 2; PCR – Polymerase chain reaction

Supplementary Table 1: Timeline of COVID-19 restrictions in Israel

DATE	Decision/Event/Limitation	CLASSIFICATION
**JAN 2020**		
24/01/2020-02/02/2020	Increasing limitations on travel to China and subsequent border closure	Travel restrictions
25/01/2020-02/02/2020	Implementation of quarantine laws for travelers returning from China	Isolation/quarantine guidelines
27/01/2020	MOH signed the People's Health Ordinance Decree	Government decision
**FEB 2020**		
16/02/2020-22/02/2020	Expansion of quarantine and isolation order to include additional Asian countries	Isolation/quarantine guidelines
23/02/2020	School delegations to Poland suspended	Activity restriction
24/02/2020	Expansion of border closure to include denial of entry from additional Asian countries	Travel restriction
27/02/2020	Italy travel warning and quarantine requirement	Travel restriction. Isolation/quarantine guidelines
**MAR 2020**		
04/03/2020	Initial guidelines including limitations on mass gatherings, isolation & quarantine for returning travelers, & travel restrictions	Travel restriction, Isolation/quarantine, Activity restriction
10/03/2020	Purim	Holiday (Jewish)
17/03/2020-19/04/2020	Full lockdown including banning gatherings over 10 people, closure of educational institutions, venues and shops, reduction of public transport, 100m radius restriction on leaving a place of residence.	LOCKDOWN
18/03/2020	Denial of entry into Israel by foreign nationals	Travel restriction
25/03/2020	Ziyarat al-Nabi Shu'ayb festivities cancelled	Holiday (Druze)
**APR 2020**		
07/04/2020	Masks required above age 6 years	Government decision
07/04/2020- 12/04/2020	No public transit or flights	Activity restriction
08/04/2020-16/04/2020	Pesach	Holiday (Jewish)
23/04/2020-10/05/2020	Closure of stores overnight in Muslim cities and towns	Activity restriction
23/04/2020-24/05/2020	Ramadan	Holiday (Muslim)
25/04/2020-26/04/2020	Allowances for outdoor prayer, weddings, mental health treatments, restaurants, street shops, cosmetic services	Activity allowance
28/04/2020-29/04/2020	Yom HaZikaron and Yom HaAtzmaut	Holiday (Israeli)
**MAY 2020**		
04/05/2020-10/05/2020	Continual opening of non-essential and leisure services, permitting of gatherings, removal of limitation on distance from place of residence, allowances to isolate at home rather than hotel facilities	Activity allowance
10/05/2020	Opening of preschools and kindergartens	Educational allowance
12/05/2020	Lag BaOmer	Holiday (Jewish)
17/05/2020	Opening of schools in accordance with local authorities' decisions	Educational allowance
24/05/2020-26/05/2020	Eid El Fitr	Holiday (Muslim)
26/05/2020	Resumption of activities such as eateries, pools and tourism	Activity allowance
**JUNE 2020**		
14/06/2020	Increase in allowances for attendees/participants of religious rites and cultural events	Activity allowance
**JULY 2020**		
02/07/2020	Limitation of permitted number of people allowed in event venues and houses of prayer	Activity restriction
**AUG 2020**		
11/08/2020	Reintroduction of cultural events in a limited capacity	Activity allowance
16/08/2020	Reopening of travel to select European countries, a “green list” countries that do not require quarantine upon entry to Israel	Travel allowance
**SEPT 2020**		
01/09/2020	School year postponed in red cities except for special education programs and programs for at-risk youth	Education restriction
08/09/2020	Nightly curfew in red cities, restricted to 500m distance from one’s place of residence, between 7pm-5am	Local travel restriction
18/09/2020-18/10/2020	Full lockdown: maximum permitted capacity for prayers over the High Holidays, 500m radius restriction on leaving one’s residence, closure of businesses and places open to the public, reduction of public transport	LOCKDOWN

18/09/2020-20/09/2020	Rosh Hashana	Holiday (Jewish)
23/09/2020	Closure of Ben Gurion airport for outgoing flights	Travel restrictions
28/09/2020	Yom Kippur	Holiday (Jewish)
**OCT 2020**		
03/10/2020-10/10/2020	Sukkot	Holiday (Jewish)
18/10/2020	Reopening of preschools and kindergartens, workplaces, restaurant takeaway services, lifting of restrictions on leaving the house, expanded allowed capacity for gatherings	Activity allowances
**NOV 2020**		
01/11/2020	Grades 1-4 return to school	Educational allowance
08/11/2020	Street-side stores permitted to reopen	Activity allowance
18/11/2020	"Green islands" in Eilat and the Dead Sea, allowances for eateries, tourism and shopping	Travel and Activity allowance
24/11/2020	Grades 5-6 return to school	Educational allowance
26/11/2020-08/12/2020	Opening of select malls and museums	Activity allowance
**DEC 2020**		
06/12/2020	Grades 10-12 return to school in green and yellow cities	Educational allowance
08/12/2020-23/12/2020	Broad opening of malls, markets and museums	Activity allowance
20/12/2020	First day of vaccinations	Vaccinations
20/12/2020	All locations considered “red”, all international passengers required to go into 14-day quarantine in hotels	Travel restriction<br>Isolation/quarantine guidelines
23/12/2020	Denial of entry into Israel from countries with new variants	Travel restriction
24/12/2020-25/12/2020	Christmas	Holiday (Christian)
27/12/2020-07/01/2021	Closure of all non-essential stores and services, 1km radius restriction on leaving one’s residence, limitations on gatherings, reduction of public transport capacity. Continued in-person education for preschool-Grade 4, Grades 11–12 & special education	LOCKDOWN
28/12/2020	Cancelation of “Green islands” designation of Eilat and the Dead Sea	Travel restriction
**JAN 2021**		
07/01/2021-07/02/2021	Further lockdown including further limitation of gatherings, educational institutions closed, permit to travel abroad limited, closure of all public and commercial establishments	LOCKDOWN (tightening)
08/01/2021-09/03/2021	Inbound passengers arriving from select countries must quarantine in hotels	Isolation/quarantine guidelines
24/01/2021-06/03/2021	Closure of Ben Gurion airport for all inbound and outbound flights.	Travel restriction
**FEB 2021**		
04/02/2021	Vaccination certificates exempting holders from quarantine	Vaccinations
07/02/2021	Reopening of workplaces, restaurant takeaway services, lifting of restrictions on leaving the house	Activity allowances
11/02/2021	Preschool-Grade 4 return to school in green, yellow and some light orange cities	Educational allowance
14/02/2021	Incoming passengers must present negative PCR test prior to departure and retest after arriving	Travel restriction
21/02/2021	Grades 5,6,11,12 return to school in green, yellow and some light orange cities	Educational allowance
21/02/2021	Green Passport for people who have received both doses of the vaccine, or recovered.<br>Broad opening of malls, markets, museums and houses of worship	Activity allowance
25/02/2021-28/02/2021	Nightly curfew 8:30pm-5:00am with travel restricted within 1km, no gatherings, business closures	Activity restriction
26/02/2021	Purim	Holiday (Jewish)
**MAR 2021**		
07/03/2021	Grades 7-10 return to school in green, yellow and some light orange cities	Educational allowance
07/03/2021	Broad reopening and varied allowances for Green Passport holders	Activity allowance
07/03/2021	Up to 3000 Israelis can enter Israel per day	Travel allowance
